# Disease-specific alterations in the expression of circulating extracellular vesicle surface proteins in sepsis, hypertension and hypertensive sepsis

**DOI:** 10.1038/s41598-026-46474-1

**Published:** 2026-05-04

**Authors:** Roushka Bhagwan-Valjee, Usri H. Ibrahim, Manu Vatish, Wei Zhang, Irene Mackraj

**Affiliations:** 1 Discipline of Human Physiology, School of Laboratory Medicine and Medical Sciences, College of Health Sciences, University of KwaZulu-Natal, Durban, South Africa; 2 Nuffield Department of Women’s & Reproductive Health (NDWRH), University of Oxford, Oxford, UK

**Keywords:** Sepsis, Hypertension, Small extracellular vesicles, Surface marker, Diagnosis, MACSPlex, Biomarkers, Cardiology, Diseases, Medical research

## Abstract

The diagnostic approach for sepsis is complicated by its pathophysiological heterogeneity and the absence of a gold-standard test. Moreover, the high prevalence of preexisting chronic diseases, specifically hypertension, further complicates clinical interpretation. Recently, extracellular vesicles (EVs) have emerged as a rich source of diagnostic biomarkers for several diseases, including sepsis and hypertension, because of their critical role in intercellular communication and inflammation. Therefore, this study aimed to explore the effects of sepsis, hypertension, and their comorbidities on the surface protein markers of small EVs (sEVs), offering a novel avenue for diagnostic discovery. sEVs were isolated from different groups (healthy controls, hypertensive, septic, and hypertensive-septic patients; *n* = 12 per group) and fully characterized, and their surface markers were identified via the MACSPlex exosome kit (a flow cytometry-based technique). Distinct profiles were identified across the hypertension, sepsis, and hypertensive-sepsis groups, revealing differentially expressed surface markers compared with healthy controls or between disease states. Our findings reveal preliminary disease-specific surface signatures under MACSPlex detection. The identification of these unique biosignatures provides a foundational framework for pioneering sEV-based diagnostic strategies. This work holds significant promise for improving patient stratification, enabling more precise biomarker-driven insights into the complex interplay of sepsis and hypertension.

**Supplementary Information:**

The online version contains supplementary material available at 10.1038/s41598-026-46474-1.

## Introduction

Sepsis is among the leading causes of mortality, with mortality rates ranging from 15–25%^1^. The most recent Global Burden of Disease (reported in 2025) analysis revealed a dramatic increase, with an estimated 166 million sepsis cases and 21.4 million deaths in 2021, representing nearly one-third of all global deaths. This reversed decades of progress, with the incidence of sepsis increasing 230% among adults since 1990^2^. The Surviving Sepsis Campaign issued the Third International Consensus Definitions for Sepsis and Septic Shock (Sepsis-3), which defines sepsis as life-threatening organ dysfunction caused by a dysregulated immune response to infection^3,4^. The pathophysiology of sepsis is highly heterogeneous and involves complex processes and various systems driven by the initial stimuli/pathogens introduced into the immune system^5–7^. The increase in pro- and anti-inflammatory pathways triggers the massive release of pathogen-associated molecules, mediators and cytokines^6,8^. This release subsequently leads to the activation of coagulation pathways and other complement cascades^7,9^. Therefore, the development of sepsis varies significantly among individuals^10^. With this, the prognosis and outcome depend on various elements specific to the individual with the illness, such as preexisting or newly developed chronic illnesses. These chronic illnesses include but are not limited to hypertension, diabetes, chronic renal disease, vascular disease, HIV and TB^10,11^. This variation still requires further elucidation to develop precise and accurate diagnostic and therapeutic tools.

Hypertension is one of the most impactful chronic illnesses that may coexist with sepsis. It is considered a potential risk factor for mortality within 28 days in sepsis patients, as revealed by a recently published study^12^. Furthermore, hypertension is one of many preexisting chronic illnesses observed in patients with sepsis^13^ and is considered more common. This, along with the fact that the number of people with hypertension doubled between 1990 and 2019, with the increase seen predominantly in low- and middle-income countries^14,15^, is the focal point. + 

A biomarker is an indicator of a disease state that can be objectively measured and monitored^16,17^. Traditional biomarkers include nucleic acids, proteins, peptides, hormones, growth factors, and antibodies^16,17^, which are used to track treatment response, enable early diagnosis, provide prognostic information, and guide therapeutic interventions^18^. Valid biomarkers must be disease-specific with minimal false positives, be minimally invasive to obtain, and be capable of informing patient prognosis and treatment outcomes^18,19^. Recently, small extracellular vesicles (sEVs) have emerged as novel and potent tools for understanding the development and progression of various diseases^20^. These small vesicles, measuring 30–200 nm in diameter, can be found in most bodily fluids^20^. sEVs play a significant role in intercellular communication, this crucial role is achieved by selecting and packaging proteins, mRNAs, miRNAs, and bioactive lipids into sEVs^21^. Given that the molecular composition of sEVs reflects the physiological state of their parent cells, extensive research has focused on their potential as biomarkers^20^.

To investigate the biomarker potential of sEVs, phenotyping of sEV groups is crucial for understanding their role in pathological states. Our previous study demonstrated that the colocalization patterns of tetraspanins (CD63, CD81, and CD9) in sepsis could serve as potential biomarkers for sepsis^22^. The functions of tetraspanins include the formation and sorting of cargo as well as the uptake and release of sEVs^23^. The structural organization of tetraspanin webs contributes to the heterogeneity of sEV surface architectures owing to their capacity to associate with other tetraspanins and membrane-bound markers^23^.

Furthermore, proteins are either encapsulated within the lumen or embedded on the surface of sEVs, enabling subtyping of these sEVs without destroying their structure^24^. These surface proteins are indicative of sEV biogenesis, secretion, protein‒protein interactions, and the targeting of recipient cells. Therefore, investigating these surface proteins in the context of sepsis pathology is crucial.

This study presents a comparative analysis of the surface marker profiles of sEVs from South African sepsis patients with or without preexisting hypertension. The primary aim of this study was to provide an understanding of the effects of preexisting hypertension on the pathophysiology of sepsis through investigating the sEV surface marker profiles associated with these disease states. To the best of our knowledge, this is the first use of a multiplexed flow cytometric bead assay (MACSPlex) in this context. This study provides important insights into the molecular distinctions between sepsis alone and sepsis complicated by hypertension. The unique surface marker signatures identified here have the potential to serve as biomarkers for diagnostic stratification and may offer future opportunities for targeted therapeutic development.

## Materials and methods

### Ethics approval

Regulatory, ethical, and institutional approvals under registration numbers BREC/00,004,587/2022 and NHRD: KZ_202107_008 were obtained from the University of KwaZulu-Natal Ethics Committee and the South African Department of Health, respectively.

### Study population and sample collection

The study population consisted of Black and Indian South African patients admitted to the intensive care units at King Edward VIII Hospital and Inkosi Albert Luthuli Hospital. All procedures done in this study were performed in accordance with the approved ethics guidelines and declaration of Helsinki regulations. Patients were recruited on the basis of the following inclusion criteria: presented with clinical manifestations consistent with sepsis and/or septic shock, were over 10 years of age, and included women who were not pregnant at the time of recruitment. The patients’ clinical characteristics are provided in a table and are presented as the means ± SDs (Supplementary Data, Table S1). The experimental study groups included patients with sepsis with previously diagnosed hypertension, including those patients on hypertensive medications except for any calcium channel blocker (*n* = 12), and a group consisting of patients with sepsis with no preexisting comorbidities (*n* = 12). This study employed two control groups, consisting of age-, sex-, and race-matched individuals with hypertension (n = 12) and a group of healthy individuals with no chronic illnesses (n = 12). Informed consent or deferred consent for all participants in this study were obtained from the patients, their next of kin or legal guardian. The treating physician took blood samples (8 mL) during the first 12 h of admission to the ICU via an arterial line. Blood samples were taken in vacutainer tubes treated with ethylenediaminetetraacetic acid (EDTA) (Becton, Dickinson and Company, SA), and plasma was obtained by centrifugation for 15 min at 3500 × g. Plasma samples were analyzed within 3 months of collection (stored at -80 °C).

### Materials

The Total Exosome Isolation Kit for plasma was purchased from Invitrogen by Life Technologies, CA, USA. The enzyme-linked immunosorbent assay for exosomal CD63, ExoELISA, was purchased from System Biosciences, Mountain View, CA. The MACSPlex Exosome Kit was purchased from Miltenyi Biotec, USA.

### sEV isolation

A Total Exosome Isolation Kit was used to isolate sEVs from the plasma samples following the manufacturer’s instructions. Briefly, clarified plasma was incubated for 10 min at 25 °C with an appropriate volume of exosome precipitation reagent and a 0.5X volume of phosphate-buffered saline (PBS). This mixture was then centrifuged for 5 min at 10,000 × g. Thereafter, the supernatant was carefully aspirated and discarded, and the sEV pellets were suspended in 0.25 mL of PBS and stored at -20 °C for future analysis.

### sEV characterization

The size distribution and concentration of isolated sEVs were evaluated via nanoparticle tracking analysis (NTA) with a NanoSight 500 NTA 3.2 NTA Release, Version Build 0069 instrument, equipped with an sCMOS camera and a Blue405 laser (Malvern Panalytical, UK). Briefly, a particle distribution of between 10 and 100 particles per image was obtained by diluting the samples in PBS. The samples were mixed to achieve uniform particle dispersion before being added to the chamber (temperature: 25 °C, viscosity: 0.86 cP). The instrument was recorded at a camera level of 12, a shutter speed of 20 ms, and a gain of 600. These parameters were held constant among all the samples. Furthermore, the morphology of isolated sEVs was analyzed via transmission electron microscopy (TEM) according to the protocol provided in our previously published study^22^.

### Quantification of sEVs

An enzyme-linked immunosorbent assay for exosomal CD63 was used to determine the abundance of sEVs among various groups according to the manufacturer’s instructions. Briefly, sEVs were incubated at 37 °C for 1 h in a microtiter plate with the binding buffer supplied in the kit. CD63 primary antibody was added after washing, and the samples were incubated for another hour. Next, another wash and incubation with the secondary antibody were performed. The plate was then rinsed and incubated with the supersensitive tetramethylbenzidine ELISA substrate at room temperature for 15 min and agitated. Finally, the absorbance was measured at 450 nm immediately after the addition of the stop buffer. The number of sEVs/ml was estimated from the CD63 signal, assuming comparable CD63 density per vesicle across groups.

### Bead-based multiplex flow cytometry assay

Isolated sEVs were subjected to a bead-based multiplex exosome flow cytometry assay following the short protocol per the manufacturer’s instructions. The input amount was 120 µl, containing approximately an equal amount of sEVs, as indicated by the CD63 ELISA results. The sample was added to a 1.5 mL tube, followed by 15 µL of MACSPlex Exosome Capture Beads (containing 39 different antibody-coated bead subsets) and 15 µL of the detection antibody cocktail (containing CD63, CD81, and CD9 antibodies). The tubes were then incubated for 1 h at room temperature in the dark on an orbital shaker at 450 rpm. Following incubation, 500 µL of MACSPlex buffer was added to each tube and centrifuged at 3000 ×*g* for 5 min at room temperature. Approximately 500 µL of the supernatant was carefully aspirated and discarded, followed by a second addition of 500 µL of the MACSPlex buffer to each tube. The tubes were incubated for 15 min at room temperature in the dark on an orbital shaker at 450 rpm, followed by centrifugation at 3000 × g for 5 min at room temperature. Approximately 500 µl of the supernatant was aspirated and discarded, and the solution was resuspended by pipetting up and down as well as by vortexing the tubes for 30 s. Flow cytometric analyses were performed with a Cytek Northern Lights flow cytometer (Cytek Biosciences B.V., Amsterdam, Netherlands). Approximately 350 µl (final sample volume diluted in MACSPlex buffer) was loaded into and acquired by the instrument, resulting in approximately 15,000 single bead events being recorded. The 39 bead populations were distinguished by the FITC and PE (excited by the blue laser) channels, and sEVs were detected via the APC (excited by the red laser) channel. Spectroflo® (Cytek Biosciences B.V., Amsterdam, Netherlands) software was used for data acquisition from each sample. FlowJo software (version 10, FlowJo LLC) was used to analyze the flow cytometric data. The median fluorescence intensity (MFI) for all 39 capture bead subsets was background-corrected by subtracting the respective MFI values from matched non-EV blank controls, which contained the sEV capture beads, antibody detection cocktail, and water, as well as the MFI of the corresponding isotype controls (mIgG1, REA). In this study, we analyzed the populations of sEVs in plasma samples from four groups (healthy controls; hypertension, sepsis and sepsis hypertension; 12 participants per group). The median fluorescence intensity (MFI) was determined, and each sample MFI was corrected with a non-EV blank control. The MFI results are presented as the means ± SEMs. Surface markers with an MFI allophycocyanin (APC) signal lower than that of the isotype controls were considered undetected.

### Statistical analysis

GraphPad Prism 10 was used to perform all the data analyses and generate graphical representations. (La Jolla, CA, USA). The statistical analysis was performed via ordinary one-way ANOVA followed by Tukey’s multiple comparisons test. Statistical significance was considered when the *p value was* < *0.05*.

## Results

### Concentration and size determination of circulating sEVs.

**Fig. 1:**
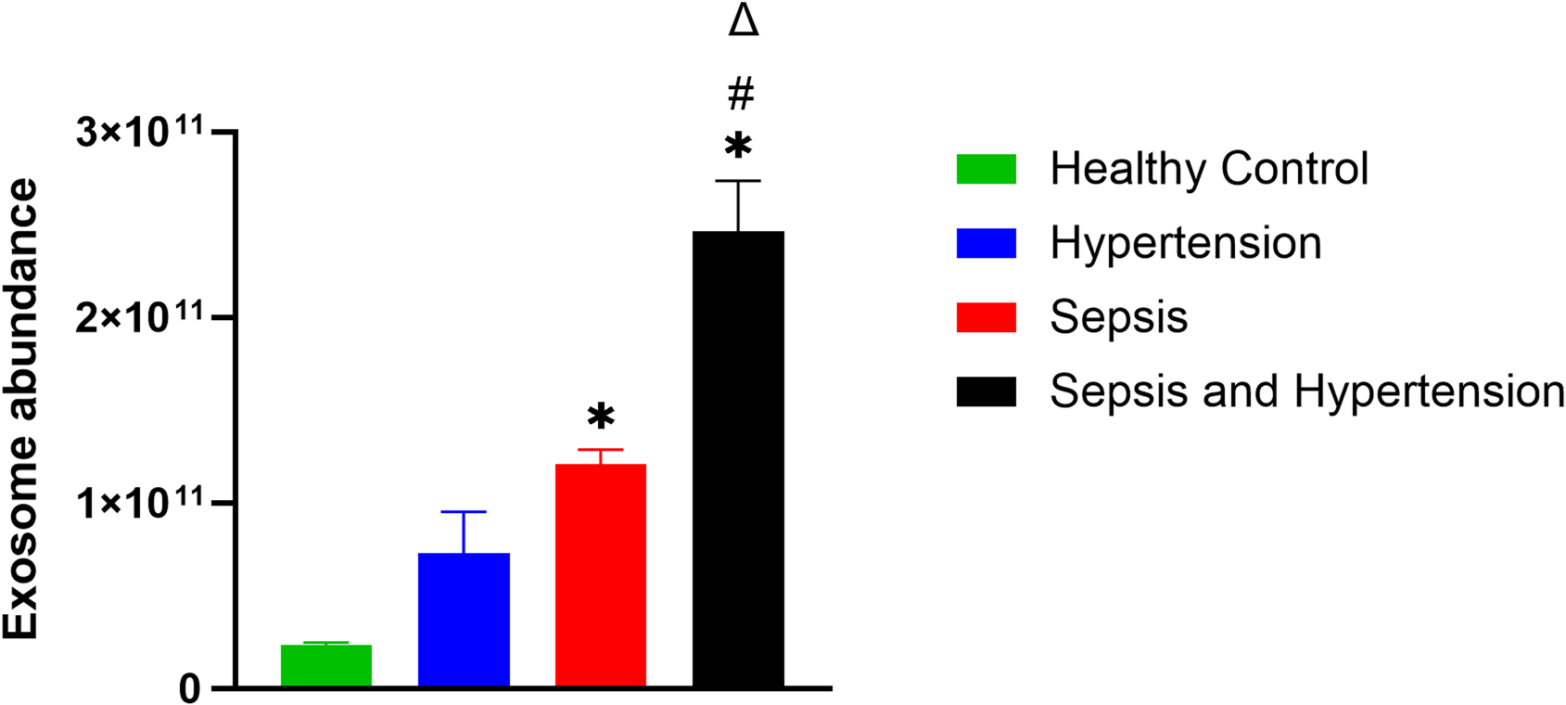
Exosome abundance/mL of plasma, determined via CD63 EXO-ELISA, among various study groups. Compared with those of healthy controls, the sepsis and sepsis hypertension groups presented significant increases (* < 0.01 and < 0.0001, respectively). Compared with the sepsis and hypertension groups, the sepsis hypertension group presented a substantial increase (# < 0.001 vs sepsis and Δ < 0.0001 vs hypertension). The data in the graphs are presented as the means ± SEMs.

To define disease-specific sEV profiles, samples were collected from patients of all groups examined in this study within the first 12 h post-admission. The healthy control and hypertensive groups consisted of volunteers who were age-, sex-, and race-matched (clinical data are provided in Table S1, supplementary data). The sEVs were isolated from plasma via a total exosome isolation kit (Invitrogen by Life Technologies, CA, USA) according to the manufacturer’s instructions to facilitate a comparative analysis among the four groups. The size distribution of the isolated sEVs was analyzed via nanoparticle tracking analysis. The isolated sEVs were within the accepted size range of 30–200 nm. Table 1 shows the average particle sizes and polydispersity indices of sEVs isolated from different groups. The average particle size of the sEVs isolated from the sepsis, hypertension and sepsis hypertension groups was significantly greater than the average particle size recorded for the healthy control group (p = 0.027, 0.028, and 0.009, respectively). No significant differences were observed among the studied groups in terms of the sEV polydispersity index. Figure S1 presents an example of the morphology of isolated sEVs, as captured via TEM. A validated CD63 ExoELISA was used to determine the exosome abundance in the isolated sEV fraction (Fig. 1), as equivalent abundances of sEVs are crucial for consistent comparisons of sEV surface markers via multiplex bead-based flow cytometric analysis^24^. The exosome abundance was significantly greater in patients with sepsis (1.21 × 10^11^ ± 1.9 × 10^10^) and sepsis with hypertension (2.46 × 10^11^ ± 6.72 × 10^10^) than in healthy controls (2.36 × 10^10^ ± 0.29 × 10^10^) (p = 0.005 and < 0.0001, respectively). Furthermore, sepsis patients with preexisting hypertension presented significantly greater levels of exosome abundance than did sepsis patients (p = 0.0005). A trend was also observed, with greater exosome abundance in hypertensive patients than in healthy controls; however, this difference was not statistically significant (p = 0.24).

**Table 1:** Average particle size and polydispersity index for sEVs isolated from the study groups. The results are presented as the means ± standard deviations, *n* = 12 per group.

Parameter	Healthy control	Hypertension	Sepsis	Sepsis hypertension
Average particle size (nm)	90.56 ± 36.52	173.06 ± 17.56	134.4 ± 2.4	162.33 ± 18.15
Polydispersity index	0.318 ± 0.076	0.231 ± 0.065	0.294 ± 0.13	0.471 ± 0.41

### Surface protein profiling of isolated sEVs

To study the surface marker profiles, isolated sEVs were analyzed via a multiplex bead-based flow cytometric assay. The MACSPlex assay enables the simultaneous, semiquantitative detection of 37 different sEV surface markers (and two internal isotope controls) in a single sample via dyed bead populations, each of which is specific to an antibody that recognizes these surface markers. Beads are differentiated by their particular fluorescence characteristics (FITC vs PE), and the binding of sEVs is detected via APC-conjugated antibodies targeting the three major tetraspanins (CD63, CD81 and CD9). The gating strategy and identification of bead populations are shown in Supplementary data Figure S2

#### EV surface marker profiles in sepsis and hypertension patients

**Fig. 2:**
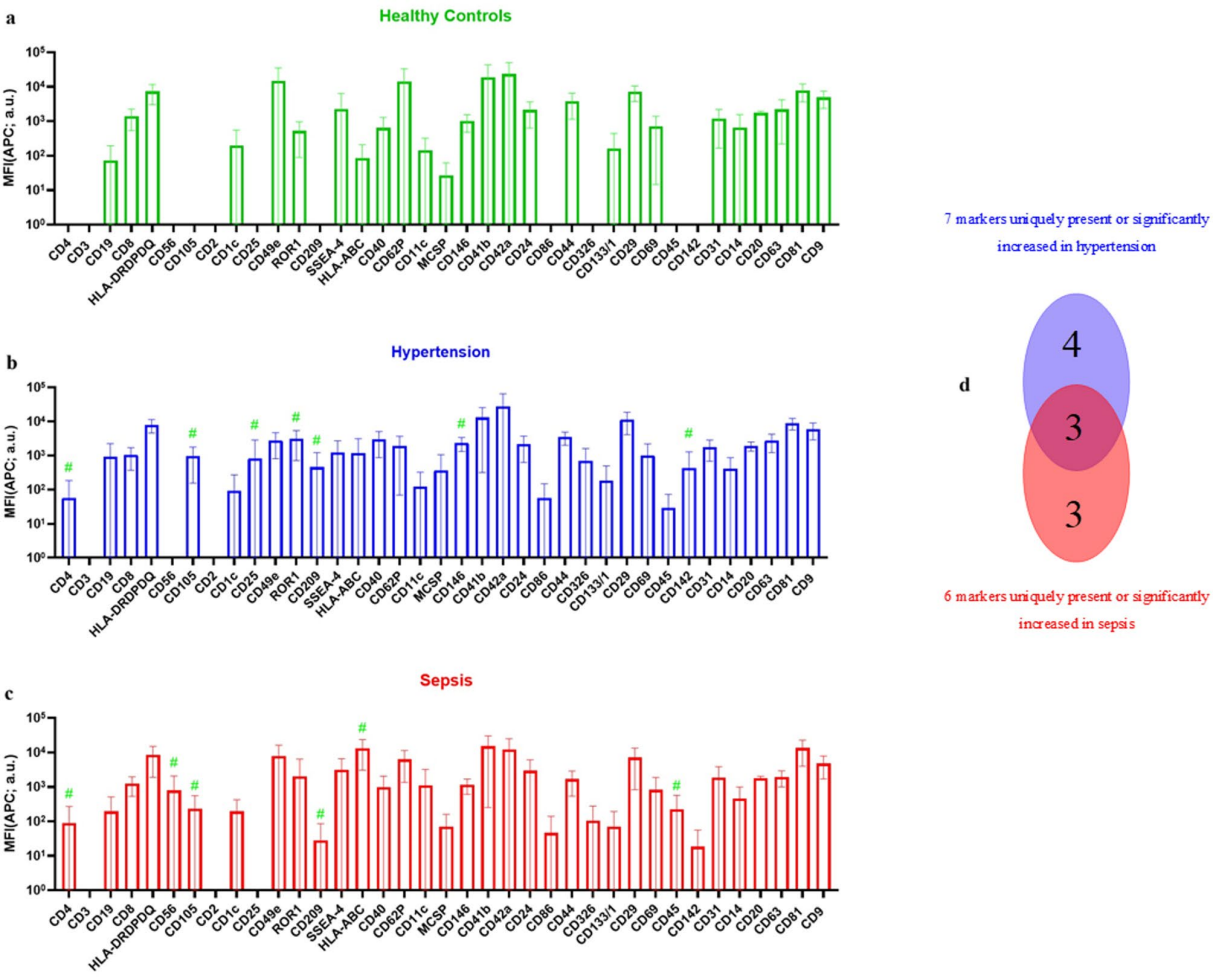
Surface marker profiles of healthy control (**a**), hypertension (**b**) and sepsis (**c**) groups (# indicates markers that are significantly increased or uniquely present in groups compared with healthy controls), *n* = 12 per group. 2d Venn diagram depicting unique and common markers in the hypertension and sepsis groups compared with the healthy control group. a.u. = arbitrary unit.

**Fig. 3:**
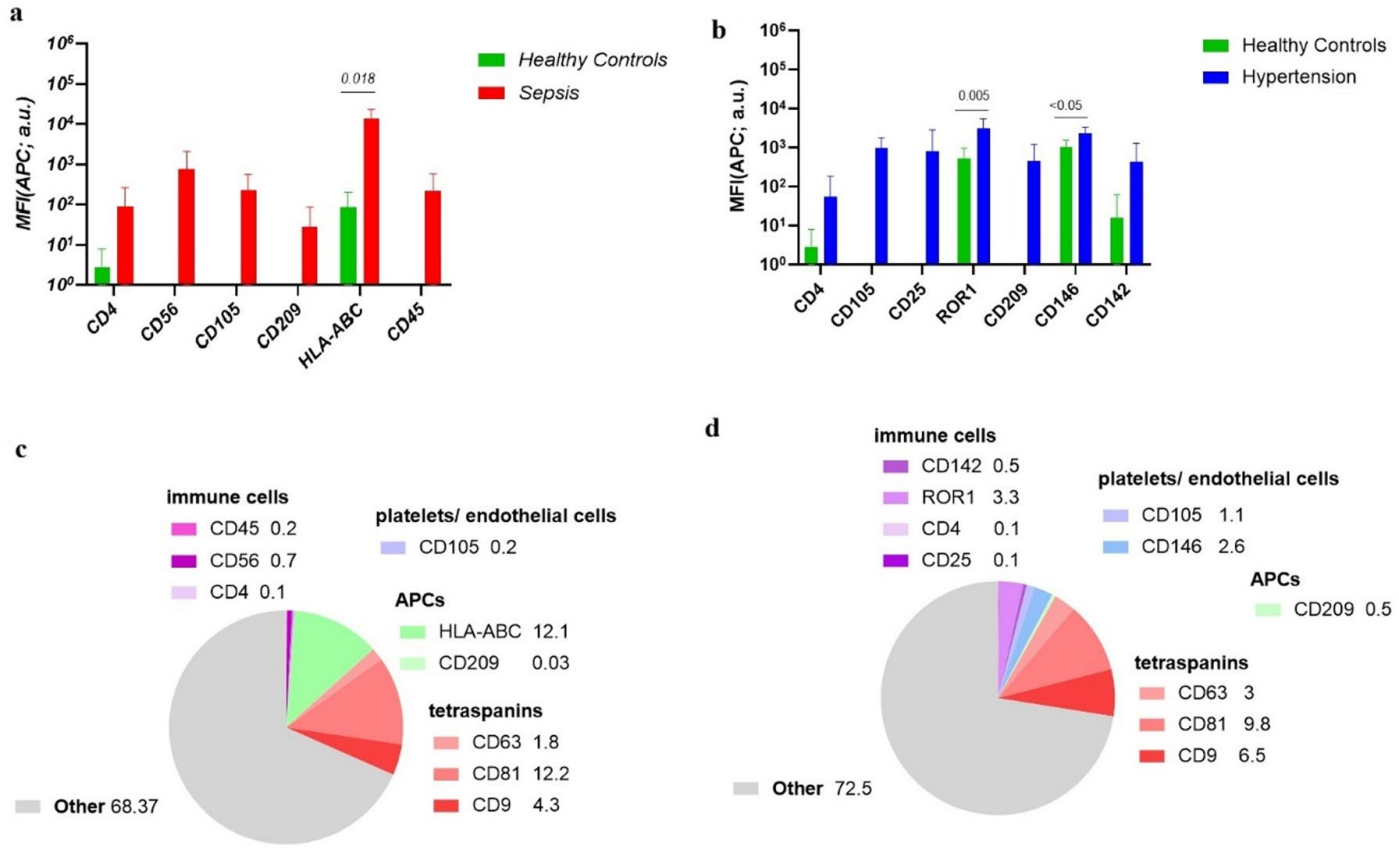
Surface markers that are differentially or uniquely expressed in the sepsis group (**a**) and hypertension group (**b**) compared with the healthy control group (n = 12 per group). Bars indicate the MFI APC signal, plotted as the mean ± SEM. Statistical significance was determined via Tukey’s multiple comparisons test. Bars without significance indicate that these surface markers were not detected in healthy controls but were present in the respective groups. Percentage expression of differentially and uniquely expressed surface markers in the sepsis group (**c**) and hypertension group (**d**), grouped according to known/potential cellular origins. “Other” in gray represents surface markers that were not significantly different or below the isotope threshold in each group. a.u. = arbitrary unit.

The results from this study, compared with the healthy control group (Fig. 2a), highlighted EV surface markers that were significantly increased or uniquely expressed in the hypertension group (Fig. 2b) and the sepsis group (Fig. 2c). A comparison of sepsis vs control and hypertension vs control revealed that six EV surface markers (CD4, CD105, CD56, HLA-ABC, CD209, and CD45; Fig. 3a) in the sepsis group and seven EV surface markers (CD4, CD105, CD25, ROR1, CD209, CD146, and CD142; Fig. 3b) in the hypertension group were significantly increased or uniquely expressed compared with those in the healthy control group. Sepsis and hypertension share three common EV surface markers (CD4, CD105, and CD209; Fig. 2d). Compared with those in healthy controls, surface markers that were not significantly increased or uniquely present in the hypertension and sepsis groups are provided in Supplementary Data Tables S2 and S3, respectively.

Thereafter, MFI values of each bead population were calculated as a percentage of the total signal to provide the percentage expression of the total signal for markers significantly increased or uniquely expressed. In this study, the total MFI signal was defined as the sum of the median fluorescence intensities of all surface markers measured within a specific experimental group (all 37 markers in the multiplex panel). To derive the percentages, assuming that all markers have equal fluorescence intensities, the MFI for each marker (after background subtraction using the isotype control) was divided by the median fluorescence intensity sum for all markers in that sample.

The surface markers were differentiated into groups on the basis of their known/potential cellular origins, namely, immune cells, platelets, endothelial cells and antigen-presenting cells (APCs), in the hypertension and sepsis groups. In the sepsis group, sEVs derived from immune cells accounted for 1% of the total MFI signal (CD4- 0.1%, CD56- 0.7% and CD45- 0.2%), whereas 0.2% of the signal from CD105 was indicative of endothelial cell-derived sEVs in sepsis. HLA-ABC and CD209 accounted for 12.7% and 0.03%, respectively, and pointed toward sEVs originating from APCs (Fig. 3c). On the other hand, 3.5% (CD4- 0.1%, CD25- 0.1% and ROR1- 3.3%) of the total MFI signal in the hypertension group was attributed to sEVs that may have originated from immune cells (T cells and B cells). The MFI signals from CD105 (1.1%), CD146 (2.6%), and CD142 (0.5%) are indicative of their cellular origins from platelets and endothelial cells, whereas those from CD209 (0.5%) suggest the presence of antigen-presenting cell-derived sEVs (Fig. 3d).

#### The effect of preexisting hypertension on the surface marker profile of sepsis patients.

**Fig. 4:**
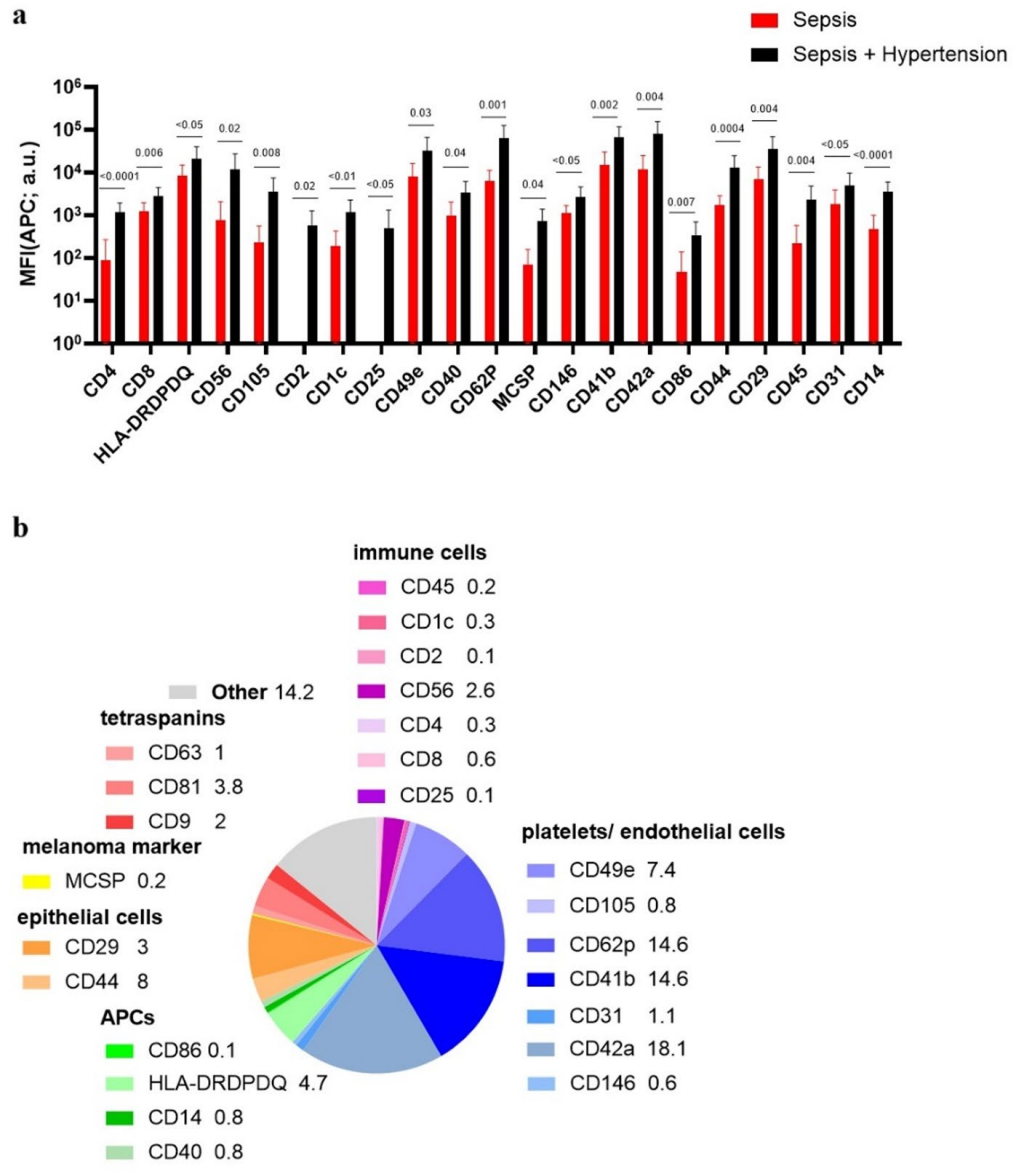
Compared with those in the sepsis group, surface markers that are differentially expressed in the sepsis with hypertension group (**a**) n = 12 per group. Bars indicate the MFI APC signal, plotted as the mean ± SEM. Statistical significance was determined via Tukey’s multiple comparisons test. Percentage expression of differentially expressed surface markers in the sepsis with hypertension group (**b**), grouped according to known/potential cellular origins. “Other” in gray represents surface markers that were not significantly different or below the isotope threshold in each group. a.u. = arbitrary unit.

Surface marker profiling of the sepsis hypertension group was performed to provide a baseline for further comparisons (Figure S3 in the supplementary data). Significant increases in the levels of twenty-one EV surface markers were recorded due to preexisting hypertension in the sepsis hypertension group. These EV surface markers include CD4; p < 0.0001, CD8; p = 0.006, CD56; p = 0.02, CD2; p = 0.02, CD1c; p < 0.01, CD25; p < 0.05, CD45; p = 0.004, CD105; p = 0.008, CD49e; p = 0.03, CD62p; p = 0.001, CD41b; p = 0.002, CD42a; p = 0.004, CD3; p < 0.05, CD146; p < 0.05, HLA-DRDPDQ; p < 0.05, CD86; p = 0.007, CD14; p < 0.0001, CD40; p = 0.04, MCSP; p = 0.04, CD44; p = 0.0004 and CD29; p = 0.004), as shown in Fig. 4a. The surface markers that were not differentially expressed between the sepsis hypertension group and the sepsis group are provided in Supplementary Data Table S4. Seventy-nine percent of the total MFI signal was attributed to the significantly increased expression of surface markers in the sepsis hypertension group compared with the sepsis group (Fig. 4b). Platelets and endothelial-derived sEVs account for 57.2% of the total MFI signal (i.e., CD49e- 7.4%, CD105- 0.8%, CD62p- 14.6%, CD41b- 14.6%, CD31- 1.1%, CD42a- 18.1% and CD146- 0.6%). Compared with those in the sepsis group, the MFI signals corresponding to CD45 (0.5%), CD1c (0.3%), CD2 (0.1%), CD56 (2.6%), CD4 (0.3%), CD8 (0.6%) and CD25 (0.1%) were indicative of the presence of sEVs derived from immune cells such as T cells, suggesting that sepsis patients with previously diagnosed hypertension have an exacerbated effect on the immune response/system. APC-derived sEVs account for 6.5% of the MFI signal (i.e., CD86- 0.1%, HLA-DRDPDQ- 4.7%, CD14- 0.8% and CD40- 0.8%), whereas sEVs originating from epithelial cells correspond to the expression of CD29 (3%) and CD44 (8%).

#### The effect of sepsis on the surface marker profile of hypertension-related EVs.

**Fig. 5:**
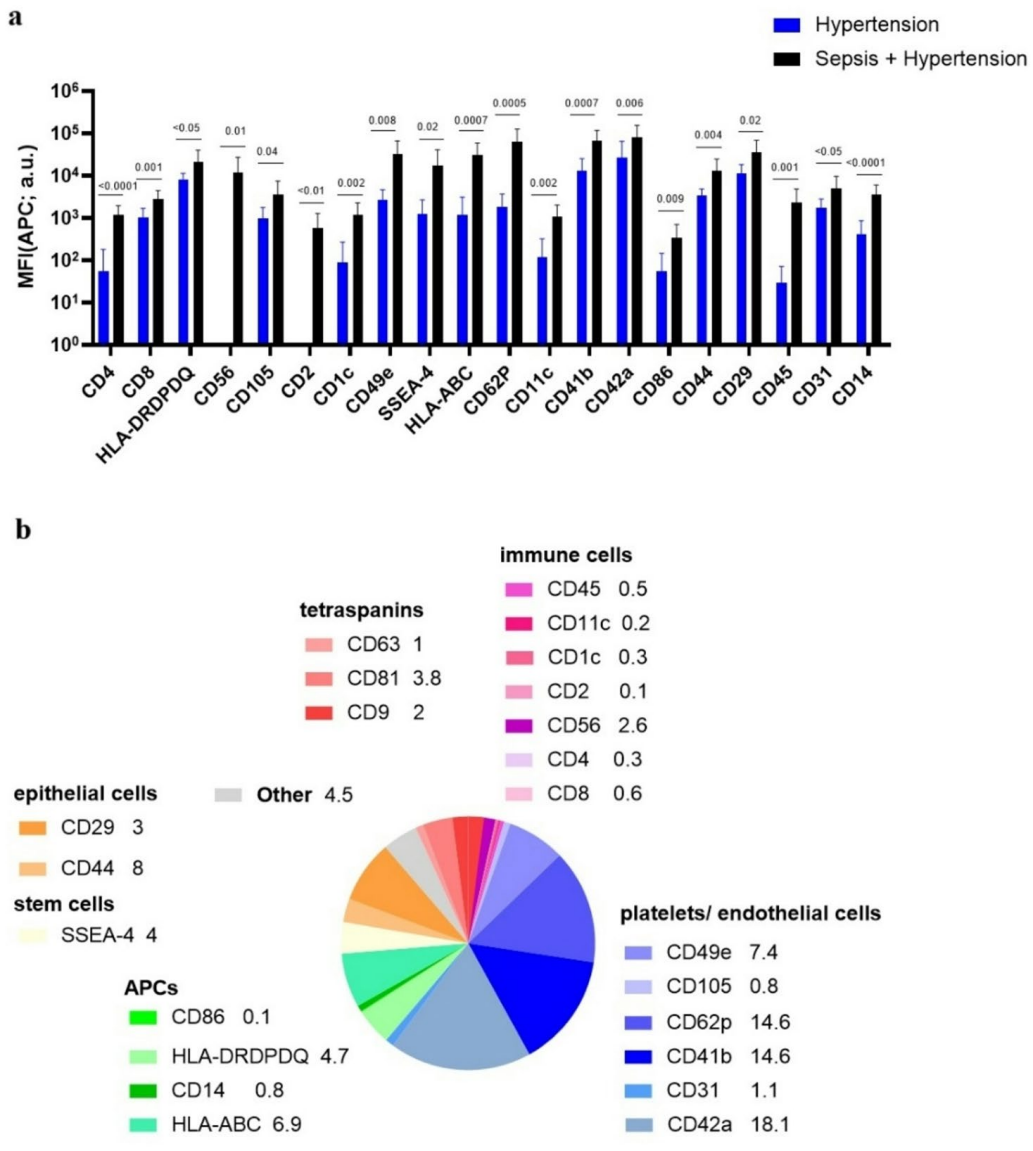
Surface markers that are differentially expressed in the sepsis with hypertension group compared with the hypertension group (**a**), n = 12 per group. Bars indicate the MFI APC signal, plotted as the mean ± SEM. Statistical significance was determined via Tukey’s multiple comparisons test. Percentage expression of differentially expressed surface markers in the sepsis with hypertension group (**b**), grouped according to known/potential cellular origins. “Other” in gray represents surface markers that were not significantly different or below the isotope threshold in each group. a.u. = arbitrary unit.

There are 37 detectable markers used in this assay, of which 20 (CD4- p < 0.0001, CD8- p = 0.001, CD56- p = 0.01, CD2- p < 0.01, CD1c- p = 0.002, CD11c- p = 0.002, CD45- p = 0.001, CD105- p = 0.04, CD49e- p = 0.008, CD62p- p = 0.0005, CD41b- p = 0.0007, CD42a- p = 0.006, CD31- p < 0.05, HLA-DRDPDQ- p < 0.05, CD86- p = 0.009, CD14- p < 0.0001, HLA-ABC- p = 0.0007, SSEA-4- p = 0.02, CD44- p = 0.004, and CD29- p = 0.02) surface markers were significantly increased in the sepsis hypertension group compared with the hypertension group, as shown in Fig. 5a. Furthermore, the percentage of the total MFI signal revealed that the cellular origins of the sEVs detected in the sepsis with hypertension group, compared with those in the hypertension group, included immune cells, platelets, endothelial cells, APCs, epithelial cells, and stem cells (Fig. 5b). The immune cell-derived sEVs are associated with MFI signals of CD45 (0.5%), CD11c (0.2%), CD1c (0.3%), CD2 (0.1%), CD56 (2.6%), CD4 (0.3%) and CD8 (0.6%), indicating that these sEVs may originate from T cells, B cells and other myeloid cells. Approximately 56.6% of the total MFI signal accounted for platelet- and endothelial-associated markers, including CD49e (7.4%), CD105 (0.8%), CD62p (14.6%), CD41b (14.6%), CD31 (1.1%) and CD42a (18.1%), suggesting that platelet-derived sEVs are highly abundant in sepsis patients with hypertension compared with hypertension patients. Moreover, sEVs corresponding to antigen-presenting cells (CD86- 0.1%, HLA-DRDPDQ- 4.7%, CD14- 0.8% and HLA-ABC- 6.9%), epithelial cells (CD29- 3% and CD44- 8%) and stem cells (SSEA-4- 4%) were detected. Overall, compared with that in the hypertension group, the MFI signal of the significantly increased surface markers in the sepsis with hypertension group accounted for 87.7% (excluding tetraspanins) of the total MFI signal. The surface markers that were not differentially expressed between the sepsis hypertension group and the hypertension group are provided in Supplementary Data Table S5.

## Discussion

The persistent challenges in diagnosing and monitoring sepsis progression have been complicated by the high prevalence of preexisting chronic illnesses, specifically hypertension, which necessitate the development of more reliable diagnostic tools that could facilitate timely and effective treatment. Recently, EVs have been introduced as an enriched source of biomarkers with the ability to overcome the limitations of current sepsis biomarkers by enabling identification of the infection source, providing a real-time snapshot of the host immune status^20^. Increasing evidence suggests that sEVs carry disease-specific molecular signatures that reflect their tissues of origin and mirror the pathophysiological state of the host. Their heterogeneous molecular composition offers unique “fingerprints” for potential biomarker development, providing valuable insights into disease processes, diagnostic stratification, and therapeutic targeting. Specifically, sepsis-associated EVs and their protein cargo have emerged as promising candidates for both diagnostic and prognostic applications^25^. Alterations in EV surface proteins have also been studied to explore their pathophysiological role and potential as biomarkers in various diseases, including cancer^26^. However, to the best of our knowledge, no studies have investigated alterations in EV surface proteins in sepsis and hypertensive sepsis, highlighting their pathophysiological role and promising diagnostic value.

This study employed the MACSPlex Exosome Human Kit (Miltenyi Biotec) to investigate disease-specific surface marker profiles of small extracellular vesicles (sEVs) in sepsis, hypertension, and sepsis complicated by preexisting hypertension. Four comparisons were conducted (healthy controls vs. sepsis patients, healthy controls vs. hypertension patients, hypertension patients vs. sepsis-induced hypertension patients, and sepsis patients vs. sepsis-induced hypertension patients) to identify the differentially expressed EV surface markers specific to each group. To our knowledge, this is the first study to characterize sEV surface markers across sepsis and hypertensive sepsis states via a multiplexed flow‐cytometric bead–based method. Herein, we discuss the EV surface markers specific to each disease, the overlap between these markers, and the effect of preexisting hypertension on the EV surface protein profile of sepsis patients.

### Sepsis-specific surface markers

This study identified six EV surface markers that were significantly elevated in sepsis compared with healthy controls. Specifically, EV-associated HLA-ABC and CD4 were significantly increased, while CD56, CD105, CD209, and CD45 showed unique expression in the sepsis group. Cell-origin annotation of these markers indicates contributions from multiple immune and endothelial cell populations. These results demonstrate that sepsis is associated with distinct alterations in the circulating sEV surface marker profile, reflecting the significant role of sEVs in immune cell signaling and proliferation, endothelial cell dysfunction and tissue damage during sepsis development and progression.

Based on the known cellular origins and functions of these markers, increased EV CD209 indicates increased activity of dendritic cells in sepsis to facilitate pathogen uptake for antigen processing and presentation^27^. Similarly, stimulation of EV CD45, CD56, HLA-ABC and CD4 expression in sepsis may induce lymphocyte, natural killer (NK) cell and helper T-cell overactivation and dysfunction, leading to lymphocytic irresponsiveness and the secretion of proinflammatory cytokines (interferon‐γ (IFN‐γ) and tumor necrosis factor‐α (TNF‐α)), resulting in tissue damage and organ failure^28–31^. The elevated presence of CD56-positive sEVs reflects NK-cell activation and may serve as an accessible surrogate marker of systemic inflammation. HLA-ABC, which is expressed on all nucleated cells, indicates broad involvement of somatic tissues in sepsis pathophysiology and is linked to the immune response and the genetic predisposition to infection^30^. On the other hand, stimulation of EV CD105 expression during sepsis alludes to the role of sepsis sEVs in the nonimmune pathology of endothelial dysfunction and coagulopathy in sepsis^32^. Overall, the overexpression of these EV proteins highlights the importance of sEVs in sepsis pathophysiology and maps the path from uncontrolled inflammation to profound immune exhaustion and organ failure.

### Hypertension-specific EV surface markers

This study revealed that hypertension induces increased expression of seven EV surface markers, three of which are shared with sepsis EV markers. In comparison, the other four EV surface markers, namely, CD146, CD25, CD142, and ROR1, were uniquely associated with hypertension. These findings indicate that hypertension is associated with distinct alterations in the circulating sEV surface marker profile, involving markers linked to immune activation, endothelial dysfunction, and coagulation-related pathways.

Based on their biological roles, increased levels of these EV surface markers indicate immune activation (CD4, CD25 and ROR1), endothelial dysfunction (CD105 and CD146), and vascular remodeling (CD145 and CD2029). CD25 and ROR1 are expressed predominantly on immune cells and play pivotal roles in the immune system^33–35^ and in cell proliferation^36^. CD25 is a marker of T-cell activation and forms the alpha chain for the interleukin-2 (IL-2) receptor, which plays a crucial role in immune homeostasis^33,35^. While ROR1 expression is typically low or absent in normal tissues, it has been linked to the pathways that lead to the generation of IL-17-producing Th17 cells. IL-17 is a key cytokine in hypertension that promotes inflammation, sodium retention in the kidney, and endothelial dysfunction^37–39^. Similarly, EV CD4 + T cells may induce the production of proinflammatory cytokines, such as IL-17, IFN-γ, and TNF-α.

On the other hand, elevated levels of EV CD146 may indicate endothelial injury^40^, as well as vascular inflammation and fibrosis^41^. Endothelial dysfunction, along with the activation of coagulation pathways, contributes to the prothrombotic state and clot formation in hypertensive patients^42,43^. CD142 (tissue factor) plays a crucial role in the activation of the blood clotting system^44^. Together, these markers highlight immune dysregulation, endothelial injury, and altered coagulation as key pathways captured in the hypertension sEV profile.

### Shared pathways: overlapping markers in hypertension and sepsis

Three surface markers, viz. CD4, CD105, and CD209 were shared between the sepsis and hypertension groups but were absent in the healthy control group. This observation indicates that these markers are shared features of the EV profiles associated with both disease states. These findings suggest that overlapping cellular pathways may underlie the EV signatures of sepsis and hypertension, implicating convergent inflammatory and endothelial pathways and organ dysfunction.

In this context, CD4, which is expressed on T cells, plays a central role in immune orchestration and the regulation of cytokines, including IL-17, which is crucial for the proliferation, differentiation and function of other immune cells, such as B cells^45^. Importantly, increases in IL-17 production and the number of IL-17-producing T cells have been reported in both sepsis and hypertension^29,46^. These increases lead to a reduced ability to mount an effective immune response when exposed to a secondary infection and the invasion of end-organ tissues^29,46^. CD209, a pathogen-recognition receptor, facilitates the initiation of immune responses and may contribute to inflammatory progression under both conditions^27^. Endothelial dysfunction is characteristic of hypertension; however, it has been suggested that stimulation of endothelial cells exacerbates inflammation through the release of proinflammatory molecules, contributing to the pathology of sepsis^47^. CD105, a component of the TGF-β receptor complex, is expressed on endothelial cells and is crucial for angiogenesis^32^. The presence of CD105 in the hypertension and sepsis groups in the present study suggests roles for CD105 in the inflammatory response and endothelial dysfunction in both diseases. These shared signatures indicate that systemic inflammation and endothelial dysfunction are core processes underpinning both diseases.

### The synergistic effect of comorbidity: sepsis with preexisting hypertension

A central finding of this study is the increased expression of multiple surface markers in sepsis patients with preexisting hypertension. Two significant comparisons were made to identify and analyze the surface marker profiles of sepsis patients in the presence of comorbidities. First, 20 out of the 37 surface markers that were increased were associated with sepsis in the preexisting hypertension group compared with the hypertension group. Second, the sepsis with preexisting hypertension vs. sepsis (with no preexisting comorbidities) group yielded 21 out of the 37 surface markers that were significantly increased. These surface markers were common to both comparisons spanning immune (CD4^48^, CD8^49^, CD56^30^, CD2^50^, CD1c^51^ and CD45^52^); platelet and endothelial functions (CD42a^53^, CD31^54^, CD41b^55^, CD62p^56^, CD105^57^, and CD49e^58^); and APC-derived sEVs (CD86^59^, HLA-DRDPDQ^60^ and CD14^61^. sEVs originating from epithelial cells include CD29^62^ and CD44^63^.

These findings strongly suggest that hypertension primes the immune and endothelial systems, rendering them hypersensitive to septic insults. The combined disease state appears to amplify immune activation, endothelial dysfunction, antigen presentation, and platelet activity-mechanisms associated with worse clinical outcomes in hypertensive septic patients, potentially increasing the vulnerability and complexity observed in these patients. The observed findings also indicate that sepsis in patients with preexisting hypertension stimulates other pathways and cells in the body. Unique signatures, including CD11c, HLA-ABC, and SSEA-4 (vs. hypertension) and CD25, CD146, CD40, and MCSP (vs. sepsis), further indicate that comorbidity introduces novel molecular phenotypes distinct from either condition alone. This molecular heterogeneity highlights the need for personalized approaches for diagnosing and managing septic patients with hypertension.

### CD56 as a candidate biomarker across the disease spectrum

CD56 was elevated in the sepsis group compared with the healthy control group and further increased in the sepsis with hypertension group compared with the sepsis group. This progressive increase suggests that CD56 may serve as a sensitive biomarker for disease severity, immune escalation, and the compounded immune dysregulation associated with hypertensive sepsis. The detection of CD2 alone in sepsis patients with hypertension suggests that additional molecular alterations associated with the hypertensive state may influence disease severity and progression.

### Mechanistic insights: platelet hyperactivation and thrombotic risk

The abundance of platelet-derived sEVs in sepsis patients with hypertension reflects the synergistic effect of platelet hyperactivation resulting from combined inflammatory and hemodynamic stressors. This massive release of procoagulant sEVs (CD62p, CD41b, and CD42a) likely amplifies thrombotic risk and could impair microcirculatory perfusion through microthrombi formation. This finding suggests an escalated prothrombotic state that may explain worse clinical outcomes in septic patients with preexisting hypertension.

Collectively, our findings suggest that the hypertension state accounts for exaggerated immune and endothelial milieu responses to septic stimuli. The sEV profile in sepsis patients with hypertension captures this synergistic dysregulation-viz. amplified immune activation, endothelial injury, and platelet hyperreactivity. These molecular insights correlate with clinical observations in septic hypertensive patients and support the diagnostic and mechanistic potential of sEV-based profiling.

### Broader implications: advancing beyond traditional diagnostic tools

Broader implications: Advancing beyond traditional complete blood counts unlike traditional hematology analyzers that rely solely on cellular and soluble factors, an integrated snapshot of systemic pathology via sEV surface markers provides stable, high-resolution information about EV biogenesis, cellular sources, and pathway activation, making them attractive for diagnostic and prognostic applications.

From a novel perspective, sEV markers are indicative of EV biogenesis, protein interactions and cell targeting and, during pathological states, have distinct compositions within their cargo and on their surface, which may serve as markers of these states. Compared with the cargo of sEVs, these markers can provide copious, stable and precise information on EV processes and pathological states, providing diagnostic and prognostic factors. Traditionally, a hematology analyzer used for complete blood counts is used for the diagnosis of sepsis. In a blood test, changes in the cellular components of the hematopoietic system or soluble factors are measured.

The current analysis and study of EVs enable the examination of signals from a broader range of sources. The analysis of surface markers on sEVs provides evidence of wide-range cell activation in sepsis. Biomarker investigations progress when the difference in a parameter is statistically significant, biologically meaningful, and clinically useful, with effect sizes and area under the curve (AUC) metrics of receiver operating characteristic curves strong enough to improve the prediction of diagnosis. The results of this study further increase the value of sEVs as diagnostic markers, in addition to the fact that these vesicles can be easily and noninvasively obtained through liquid biopsies. However, due to limitations of this study, the extent to which these sEV markers directly reflect common pathogenic mechanisms remains a matter of mechanistic interpretation and requires further experimental and clinical validation.

## Conclusion

This study identified disease-associated alterations in sEV surface protein profiles across patients with sepsis, hypertension and sepsis with preexisting hypertension. The findings of this study provide preliminary information on the effect of these pathophysiological states on the heterogeneity of the circulating sEVs and their cellular origin, highlighting the potential of these variations as promising diagnostic, prognostic and patients stratification tools for sepsis, hypertension and hypertensive sepsis. Furthermore, this study identified a unique association between EV CD56 and CD45 with sepsis, EV CD146 and CD142 with hypertension, and marked increase in platelet-derived sEVs in hypertensive sepsis patients. Moreover, preexisting hypertension in sepsis patients significantly elevated the expression of 17 common EV surface proteins compared with those in patients with either condition alone underscore the synergistic pathophysiological impact of these comorbidities on EV surface protein profiles. However, due to the exploratory nature of the study and the limited sample size, these associations remain preliminary and require further validation.

Study limitations include the small sample size, suboptimal methods of sEVs isolation and quantification of surface markers. Therefore, future validation using optimal methods for sEVs isolation and quantification of surface markers in larger cohorts, and integration of temporal profiling, and functional characterization of identified markers are highly recommended.

## Supplementary Information

Below is the link to the electronic supplementary material.

Supplementary Material 1

## Data Availability

The authors confirm that the data supporting the findings of this study are available within the article.
