# Supplementary material for: Disease-specific alterations in the expression of circulating extracellular vesicle surface proteins in sepsis, hypertension and hypertensive sepsis
Source: Sci Rep. 2026 May 4;16:20470. doi: 10.1038/s41598-026-46474-1 (PMC13328539; doi:10.1038/s41598-026-46474-1)
Supplement: Supplementary file 1 — Supplementary Material 1 [file 41598_2026_46474_MOESM1_ESM.docx]

**Supplementary Data**

**Characterizing the surface marker profiles of circulating small extracellular vesicles in sepsis, hypertension and sepsis with hypertension.**

**Roushka Bhagwan-Valjee^1^, Usri H. Ibrahim^1#^, Manu Vatish^2^, Wei Zhang^2^, Irene Mackraj^1#^**

^1^ Discipline of Human Physiology, School of Laboratory Medicine and Medical Sciences, College of Health Sciences, University of KwaZulu-Natal, Durban, South Africa.

^2^ Nuffield Department of Women's & Reproductive Health (NDWRH), University of Oxford, Oxford, United Kingdom

**^#^** Corresponding authors.

Email address: [Mackraji@ukzn.ac.za](mailto:Mackraji@ukzn.ac.za), [Usrihasan@yahoo.com](mailto:roushkab@gmail.com%20)


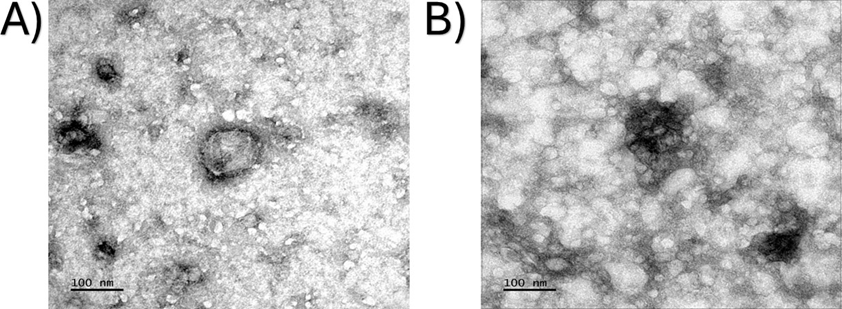
**Figure S1.** TEM analysis shows an example of the morphology of isolated sEVs. Spherical vesicles with an approximate size range of 30-150nm were observed.

**
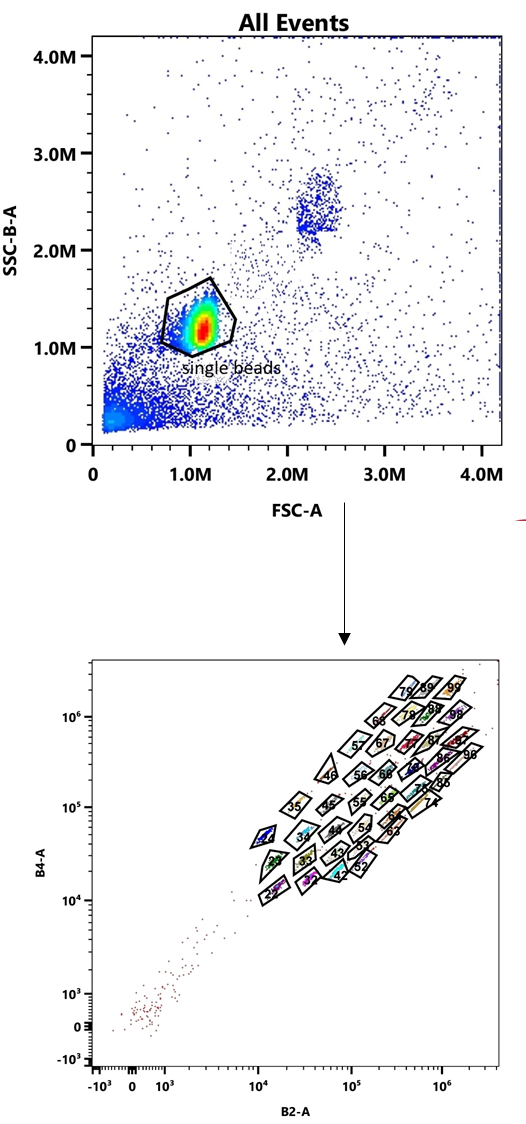
Figure S2**. The gating strategy, applied to gate single beads, is followed by additional gates to identify the 39 distinct capture bead populations, as shown. All bead populations were identified according to the manufacturer’s recommendations.


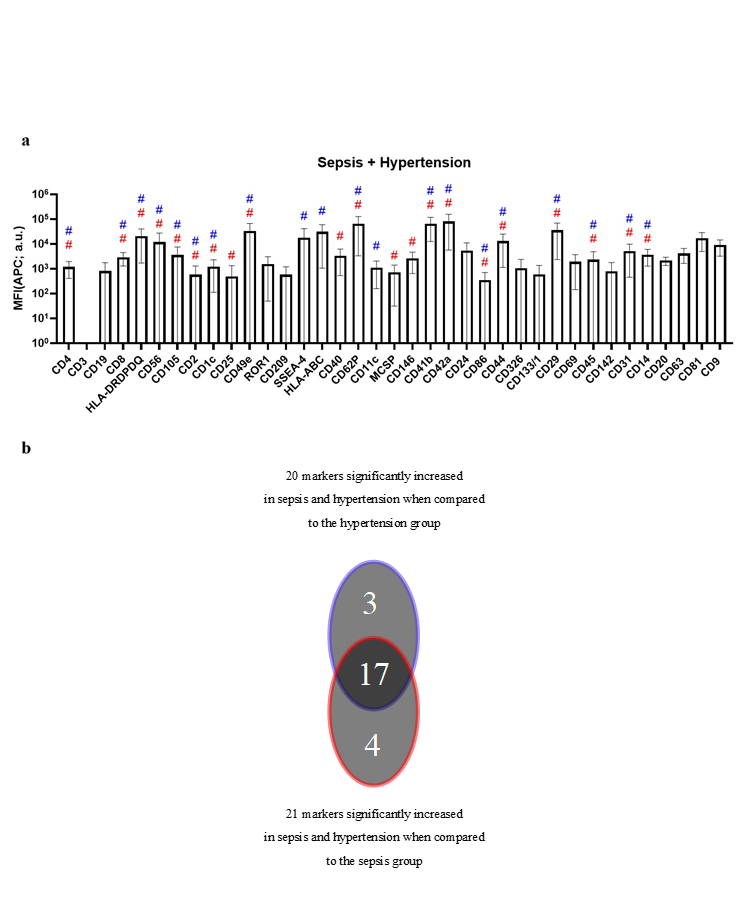


**Figure S3.** Surface marker profile of sepsis with hypertension group (a) n=12. Markers that are significantly increased when compared to the hypertension group (depicted by #) and the sepsis group (depicted by #). 2b Venn Diagram depicting unique and common markers in the sepsis with hypertension group when compared to the hypertension and sepsis groups. a.u = arbitrary unit.

**Table S1** Clinical characteristics of participants

| Variables | Sepsis Patients | Sepsis patients with hypertension |
| --- | --- | --- |
| Age range | *20-71 years* | *31-84 years* |
| Race | *Indian (50%)*  *African (50%)* | *Indian (30%)*  *African (70%)* |
| SOFA score range | *2-12* | *4-15* |
| Blood pressure (mmHg) | *Systolic 118±15.8*  *Diastolic 75±17.3* | *134±20.3*  *87±10.8* |
| Heart rate (beats/min) | *133±25.58* | *102±23.32* |
| Haemoglobin (g/mol) | *10.02±1.72* | *8.27±1.71* |
| Respiratory rate (breaths/min) | *22.83±8.12* | *19.67±6.12* |
| O_2_ saturation (%) | *98.75±1.55* | *96.92±6.76* |
| C-Reactive Protein (µg/mL) | *193.58±114.61* | *195.33±105.95* |
| White cell count (x10⁹per L blood) | *14.22±10.81* | *12.92±8.18* |
| Platelet count (x10^3^ /µL) | *129.83±75.82* | *169.42±108.89* |
| Bilirubin (mg/dL) | *39.35±92.43* | *14.25±13.77* |
| Creatinine (mg/dL) | *106.25±68.61* | *319.1±254.95* |
| Mean arterial pressure (mmHg) | *89.42±20.55* | *83.58±18.30* |

**Notes:** All values are represented as the mean±SD

| Markers | MFI (APC; a.u.) | | P-value |
| --- | --- | --- | --- |
|  | **Control (n=12)** | **Hypertension (n=12)** |  |
| CD3 | 0 | 0 | - |
| CD19 | 71,6±40,2 | 909,0±399,0 | 0.15 |
| CD8 | 1393,7±259,8 | 1026,3±208,0 | 0.85 |
| HLA-DRDPDQ | 7341,7±1425,3 | 7958,4±1073,5 | 0.99 |
| CD56 | 0 | 0 | - |
| CD2 | 0 | 0 | - |
| CD1c | 197,6±112,7 | 90,3±59,8 | 0.98 |
| CD49e | 14720,2±6202,7 | 2752,0±618,3 | 0.54 |
| SSEA-4 | 2300,9±1367,1 | 1240,7±459,4 | 0.99 |
| HLA-ABC | 86,2±49,6 | 1168,8±618,1 | 0.99 |
| CD40 | 642,6±246,8 | 2974,4±745,0 | 0.09 |
| CD62P | 14345,3±5461,3 | 1874,2±571,0 | 0.83 |
| CD11c | 143,8±53,7 | 121,3±61,2 | 0.99 |
| MCSP | 26,7±11,6 | 360,3±218,9 | 0.5 |
| CD41b | 18833,8±7818,7 | 6357,7±1249,8 | 0.83 |
| CD42a | 23534,2±8520,5 | 17468,1±5246,3 | 0.99 |
| CD24 | 2129,2±451,8 | 2175,4±468,0 | >0.99 |
| CD86 | 7,9±7,9 | 55,6±29,0 | 0.95 |
| CD44 | 3828,2±806,6 | 3448,5±435,3 | 0.99 |
| CD326 | 50,1±50,1 | 681,6±304,2 | 0.41 |
| CD133/1 | 162,5±88,8 | 180,2±94,2 | 0.99 |
| CD29 | 7156,1±1143,4 | 11350,5±2294,0 | 0.96 |
| CD69 | 712,1±220,6 | 980,0±358,4 | 0.96 |
| CD45 | 0 | 28,9±13,7 | >0.99 |
| CD31 | 1189,6±341,4 | 1754,8±358,6 | 0.97 |
| CD20 | 1748,7±61,3 | 1910,2±173,7 | 0.89 |
| CD14 | 660,6±269,9 | 403,5±147,2 | 0.97 |
| CD63 | 2223,8±603,9 | 2716,7±454,1 | 0.92 |
| CD81 | 7940,0±1424,2 | 8989,5±1065,3 | 0.99 |
| CD9 | 4931,5±809,9 | 5923,4±921,3 | 0.93 |

**Table S2** The median fluorescent intensity (MFI) of markers expressed in the control group vs hypertensive group. These markers are not considered significant as p>0.05. Data is expressed as mean ± SEM

**Table S3.** The median fluorescent intensity (MFI) of markers expressed in the control group vs sepsis patients. These markers are not considered significant as p>0.05. Data is expressed as mean ± SEM

| Markers | MFI (APC; a.u.) | | P-value |
| --- | --- | --- | --- |
|  | **Control (n=12)** | **Sepsis (n=12)** |  |
| CD3 | 0 | 0 | - |
| CD19 | 71,6±40,2 | 196,6±100,5 | 0.99 |
| CD8 | 1393,7±259,8 | 1253,8±227,3 | 0.99 |
| HLA-DRDPDQ | 7341,7±1425,3 | 8451,8±1972,2 | 0.99 |
| CD2 | 0 | 41,4±41,4 | 0.99 |
| CD1c | 197,6±112,7 | 194,0±78,5 | >0.99 |
| CD25 | 0 | 0 | - |
| CD49e | 14720,2±6202,7 | 7955,6±2533,5 | 0.86 |
| SSEA-4 | 2300,9±1367,1 | 3170,0±1147,0 | 0.99 |
| CD40 | 642,6±246,8 | 980,3±323,6 | 0.98 |
| CD62P | 14345,3±5461,3 | 6347,7±1579,2 | 0.95 |
| CD11c | 143,8±53,7 | 491,8±200,4 | 0.50 |
| MCSP | 26,7±11,6 | 70,6±29,9 | 0.99 |
| CD146 | 1012,9±168,4 | 1150,3±179,6 | 0.99 |
| CD41b | 18833,8±7818,7 | 15266,5±4334,2 | 0.99 |
| CD42a | 23534,2±8520,5 | 12157,1±4136,6 | 0.94 |
| CD24 | 2129,2±451,8 | 2980,1±913,8 | 0.93 |
| CD86 | 7,9±7,9 | 46,8±29,6 | 0.97 |
| CD44 | 3828,2±806,6 | 1709,3±336,9 | 0.85 |
| CD326 | 50,1±50,1 | 104,8±58,9 | 0.99 |
| CD133/1 | 162,5±88,8 | 69,9±39,0 | 0.97 |
| CD29 | 7156,1±1143,4 | 7099,7±1889,9 | >0.99 |
| CD69 | 712,1±220,6 | 829,5±319,9 | 0.99 |
| CD142 | 15,6±15,6 | 18,4±12,4 | >0.99 |
| CD31 | 1189,6±341,4 | 1866,1±611,1 | 0.94 |
| CD20 | 1748,7±61,3 | 1808,3±79,5 | 0.99 |
| CD14 | 660,6±269,9 | 468,3±166,7 | 0.99 |
| CD63 | 2223,8±603,9 | 1969,5±292,2 | 0.99 |
| CD81 | 7940,0±1424,2 | 13480,7±2853,8 | 0.43 |
| CD9 | 4931,5±809,9 | 4792,5±929,2 | 0.99 |

**Table S4.** The median fluorescent intensity (MFI) of markers expressed in sepsis patients vs patients with sepsis and hypertension. These markers are not considered significant as p>0.05. Data is expressed as mean ± SEM

| Markers | MFI (APC; a.u.) | | P-value |
| --- | --- | --- | --- |
|  | **Sepsis (n=12)** | **Sepsis and Hypertension (n=12)** |  |
| CD3 | 0 | 0 | - |
| CD19 | 196,6±100,5 | 811,7±282,7 | 0.37 |
| ROR1 | 748,3±205,4 | 1549,8±500,3 | 0.58 |
| SSEA-4 | 3170,0±1147,0 | 17739,8±7350,4 | 0.06 |
| HLA-ABC | 13382,0±3635,2 | 30620,8±8534,7 | >0.99 |
| CD11c | 491,8±200,4 | 1089,3±294,4 | 0.11 |
| CD24 | 2980,1±913,8 | 5410,9±1766,8 | 0.33 |
| CD326 | 104,8±58,9 | 1043,1±398,9 | 0.09 |
| CD133/1 | 69,9±39,0 | 586,7±247,6 | 0.06 |
| CD69 | 829,5±319,9 | 35697,9±10056,8 | 0.21 |
| CD142 | 18,4±12,4 | 803,3±292,8 | 0.06 |
| CD20 | 1808,3±79,5 | 2126,3±234,1 | 0.54 |
| CD63 | 1969,5±292,2 | 4109,5±745,4 | 0.04 |
| CD81 | 13480,7±2853,8 | 16840,1±3964,1 | 0.79 |
| CD9 | 4792,5±929,2 | 8919,2±1802,7 | 0.08 |

**Table S5.**  The median fluorescent intensity (MFI) of markers expressed in the hypertensive group vs patients with sepsis and hypertension. These markers are not considered significant as p>0.05. Data is expressed as mean ± SEM

| Markers | MFI (APC; a.u.) | | P-value |
| --- | --- | --- | --- |
|  | **Hypertension (n=12)** | **Sepsis and Hypertension (n=12)** |  |
| CD3 | 0 | 0 | - |
| CD19 | 909,0±399,0 | 811,7±282,7 | 0.99 |
| CD25 | 56,1±56,1 | 492,1±265,9 | 0.16 |
| ROR1 | 3053,0±828,1 | 1549,8±500,3 | 0.14 |
| CD40 | 2974,4±745,0 | 3372,1±948,8 | 0.97 |
| MCSP | 360,3±218,9 | 720,4±207,8 | 0.38 |
| CD146 | 2343,0±312,0 | 2649,6±634,4 | 0.94 |
| CD24 | 2175,4±468,0 | 5410,9±1766,8 | 0.13 |
| CD326 | 681,6±304,2 | 1043,1±398,9 | 0.78 |
| CD133/1 | 180,2±94,2 | 586,7±247,6 | 0.18 |
| CD69 | 980,0±358,4 | 1925,9±594,1 | 0.33 |
| CD142 | 431,4±267,8 | 803,3±292,8 | 0.59 |
| CD20 | 1910,2±173,7 | 2126,3±234,1 | 0.77 |
| CD63 | 2716,7±454,1 | 4109,5±745,4 | 0.29 |
| CD81 | 8989,5±1065,3 | 16840,1±3964,1 | 0.17 |
| CD9 | 5923,4±921,3 | 8919,2±1802,7 | 0.28 |
